# New targets in diabetic retinopathy: addressing limitations of current treatments through the Sema3A/Nrp1 pathway

**DOI:** 10.1038/s41433-025-03835-w

**Published:** 2025-07-09

**Authors:** Sobha Sivaprasad, Chui Ming Gemmy Cheung, Martin Gliem, Charles C. Wykoff, Nina Zippel, Susumu Ishida, Quan Dong Nguyen

**Affiliations:** 1 National Institute of Health Research Biomedical Research Centre, Moorfields Eye Hospital NHS Foundation Trust, London, UK; 2 University College London Institute of Ophthalmology, London, UK; 3 SingHealth Research, Singapore, Singapore; 4 Boehringer Ingelheim International GmbH, Ingelheim am Rhein, Germany; 5 Retina Consultants of Texas, Blanton Eye Institute, Houston Methodist Hospital, Houston, TX USA; 6 Boehringer Ingelheim Pharma GmbH & Co. KG, Biberach an der Riß, Germany; 7 Department of Ophthalmology, Faculty of Medicine and Graduate School of Medicine, Hokkaido University, Sapporo, Japan; 8 Byers Eye Institute, Stanford University School of Medicine, Palo Alto, CA USA

**Keywords:** Retinal diseases, Eye diseases

## Abstract

**Abstract:**

Diabetic retinopathy (DR) is a leading cause of acquired blindness. Retinal non-perfusion (RNP) is associated with DR worsening and vision loss. There are no treatments available that specifically address RNP in DR. The semaphorin 3A (Sema3A)/neuropilin 1 (Nrp1) pathway may be involved in RNP progression in DR. In DR, capillary dropout leads to RNP, subsequent hypoxia and ischaemia. Upon chronic hypoxia, retinal cells produce various factors, including vascular endothelial growth factor (VEGF) and Sema3A. While VEGF promotes the growth of new vessels, elevated Sema3A forms a chemical barrier in the retina that directs new blood vessels away from the ischaemic retina. The imbalance of VEGF and Sema3A in DR is believed to dysregulate physiological revascularisation in the retina and may guide blood vessels away from ischaemic regions into the vitreous cavity, causing the pathological neovascularisation typically found in advanced DR. Approved treatments can improve DR severity, but do not appear to improve the underlying RNP. This may lead to a high treatment burden over time and a risk for disease worsening once therapy is stopped, as the underlying disease may progress despite treatment. Therapeutic agents targeting the Sema3A/Nrp1 pathway may have the potential to improve RNP as a core pathophysiologic aspect of DR. This potential disease-modifying effect may sustainably improve DR and preserve the patient’s visual function and quality of life. This review summarises Sema3A/Nrp1 pathway involvement in DR and RNP and its role as a potential target to treat DR in the context of current treatment options.

**Method oF Literature Search:**

Background literature was searched in PubMed using search terms such as ‘diabetic retinopathy’, ‘diabetic macular ischemia’, ‘diabetic macular edema’, ‘semaphorin 3a’, ‘neuropilin 1’, ‘retinal non-perfusion’, ‘vascular perfusion’, ‘anti-VEGF’, ‘corticosteroid’ and ‘laser photocoagulation’. Selected articles in English included the following publication types: Clinical Study; Clinical Trial; Clinical Trial, Phase I; Clinical Trial, Phase II; Clinical Trial, Phase III; Clinical Trial, Phase IV; Clinical Trial Protocol; Controlled Clinical Trial; Meta-Analysis, Randomised Controlled Trial; Review; and Systematic Review. Reference lists from the selected articles were also reviewed, from which relevant articles were manually included into the final list, in addition to extensive general background reading about the topic. Additionally, ClinicalTrials.gov and Google searches were performed to identify upcoming trials of treatments in DR with the potential to improve RNP.

**Plain Language Summary:**

The retina is the light-sensing layer at the back of the eye. Damage to the retina can lead to eye diseases. Diabetes can cause reduced blood flow to the retina, damaging the retina and leading to vision loss. Eye disease is common in people with diabetes. This review discusses different drugs currently used to treat sight loss in people with diabetes. Taking some of these drugs can affect the quality of a person’s life. For example, the treatment may need to be given by regular injections into the eye and/or may have upsetting side effects. This review also talks about new drugs now being studied to improve blood flow to the retina and slow or stop sight loss in people with diabetes. Proteins called semaphorin 3A and neuropilin 1 regulate blood flow to the retina. New drugs have been made that work against these proteins. These drugs may help to increase blood flow to the retina. Several other drugs that may increase blood flow to the retina are also being studied in animals and humans.

## Introduction

Diabetic retinopathy (DR) is a common complication of diabetes mellitus and a leading cause of acquired blindness in working-age adults with diabetes; DR is estimated to affect approximately a third of patients with diabetes worldwide [1]. As non-proliferative DR (NPDR) advances to proliferative DR (PDR), retinal neovascularisation develops, and serious complications may arise, including retinal and vitreous haemorrhages and tractional retinal detachment, which can lead to substantial vision impairment [2]. Currently approved treatments for DR generally target late‑stage disease, at which point irreversible damage to retinal tissue has often occurred [3].

Many of the cellular and clinical alterations associated with DR result in a breakdown of the blood-retinal barrier and loss of normal retinal vasculature [4]. This creates areas of non-perfusion in the retina known as retinal non-perfusion (RNP) [4]. RNP manifests early in DR, even in eyes that do not show any evidence of clinical DR [4]. Although it is most frequently found in the retinal periphery [5, 6], RNP can also affect the central retina (macula), and in the latter case, the term diabetic macular ischaemia (DMI) is also used [7]. RNP is an independent risk factor for DR progression [6], but it can also directly affect the function of retinal cells, which can be of particular importance if RNP is located centrally in the macula [7]. Indeed, RNP has been associated with reduced retinal sensitivity on microperimetry, deterioration of best corrected visual acuity and a risk of developing vision-threatening complications [8]. RNP can substantially affect an individual patient’s vision, although the exact association between RNP, ischaemia and loss of function remains poorly understood [9].

Areas of RNP usually cannot be easily seen on fundoscopic or colour fundus imaging, and the visualisation of RNP requires dedicated imaging techniques [4]. Fluorescein angiography remains the diagnostic standard for the identification of RNP, and advancements in ultra-widefield angiographic techniques enable more comprehensive examination of peripheral retinal areas [10]. Angiography only allows for two-dimensional imaging of the retinal vasculature, requires skilled technicians for good imaging quality, and the intravenous injection of the dye may cause allergic reactions in some patients [10]. Areas of RNP are usually defined as areas with capillary loss leading to reduced fluorescence in the affected areas [11]. The analysis of RNP requires well-trained personnel because various factors can impact the visualisation of areas of RNP, including image quality, timing of the dye injection and eccentricity changes [12–14].

Optical coherence tomography angiography (OCTA), a technique that allows three-dimensional assessment of the retinal vasculature, is an alternative to fluorescein angiography [10]. Advantages of OCTA include its non-invasiveness, reproducibility and the better contrast of areas of RNP compared with the surrounding areas [10, 14]. However, the availability of OCTA is limited, and visualisation is focused on the central retina, because the imaging of peripheral retinal areas using OCTA is difficult [10]. Visualisation of non-perfusion in the peripheral retina can be achieved using swept-source OCTA, which allows a wider field of vision, but experience with this new technique remains limited.

In general, there is a lack of consensus on the optimal imaging biomarkers for RNP, although fluorescein angiography and OCTA are well-established techniques. The most common methods of assessing RNP on fundus fluorescein angiography involve measuring the total area of RNP or calculating the non-perfusion index (total area of RNP divided by gradable retinal area) [6, 10]. However, there is no published consensus on the exact definition of RNP and whether to use specific overlayed grids to assess the topography of measurements. Metrics derived from OCTA focusing on the central retina include vessel density, size of the foveal avascular zone and fractal dimension, and these may function as surrogates for the measurement of peripheral RNP [15]. Overall, there is a need for a consensus on imaging techniques and biomarkers for RNP. This may also include the application of automated algorithms to standardise the analysis of RNP from retinal images.

**Fig. 1:**
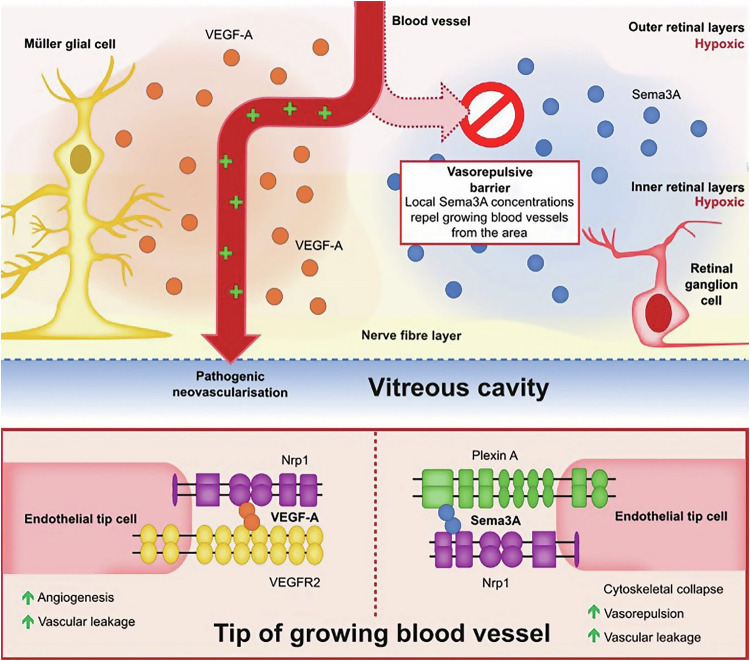
Sema3A and VEGF signalling via Nrp1 in angiogenesis and vascular permeability in DR. Created using information from Joyal et al. [17], Raimondi et al. [18], Goldman et al. [19] and Ochsenbein et al. [20]. DR diabetic retinopathy, Nrp1 neuropilin 1, Sema3A semaphorin 3A, VEGF vascular endothelial growth factor, VEGFR2 VEGF receptor 2.

Preclinical data have identified semaphorin (Sema) proteins and their co-receptors (neuropilin [Nrp] and plexins) as key regulators of morphology and motility in many different cell types, including in the nervous, cardiovascular, immune, endocrine, hepatic, renal, reproductive, respiratory and musculoskeletal systems [16–21]. Semaphorins were initially characterised by their role in axonal guidance and subsequent development of the nervous system [22]. However, Sema3 proteins (Sema3) are now gaining increasing attention for their key role in vascular guidance [22]. Sema3 proteins inhibit physiological neovascularisation and promote pathological neovascularisation via vascular endothelial cells and macrophages, respectively (Fig. 1) [16–20, 23]. In particular, Sema3A has been shown to exhibit vasorepulsive effects, predominantly via plexin A‑mediated cytoskeletal collapse, leading to impaired migration and proliferation of vascular endothelial cells [22]. Therefore, Sema3A is important in diseases characterised by angiogenesis, such as cancer.

A negative relationship between Sema3A and matrix metalloproteinase (MMP) enzymes, which facilitate tumour invasion and metastasis, has been demonstrated in cancer [24, 25]. Specifically, research has highlighted that the degradation of perlecan-Sema3A-PlexinA1-Nrp1 receptor complexes on prostate cancer cells by MMP7 regulates tumour cell migration by destabilising cell junctions [24, 25].

The role of Sema3A in directing angiogenesis is also important in the context of DR. In individuals with DR, Sema3A is secreted by hypoxic neurons in the avascular retina in response to proinflammatory cytokine interleukin 1 beta [17]. Sema3A promotes vascular decay, inhibiting normal physiological revascularisation and forming a chemical barrier that forces angiogenesis towards the vitreous, where neovascularisation is pathological [17]. Therefore, silencing retinal Sema3A/Nrp1 signalling represents a potentially important therapeutic target for patients with RNP, because doing so could redirect angiogenesis towards the retina, ultimately leading to repair of the ischaemic tissue and reduced pathophysiological neovascularisation in the vitreous [17]. As such, Sema3A may be of major clinical relevance to the management of DR in the future.

This review describes the Sema3A/Nrp1 pathway and its relation to ischaemia, summarises the impact and limitations of current therapies for DR and diabetic macular oedema (DMO) on RNP, and discusses potential therapeutic strategies targeting the Sema3A/Nrp1 pathway, which may address the unmet need of improving RNP.

## Structural changes in DR and RNP

**Fig. 2:**
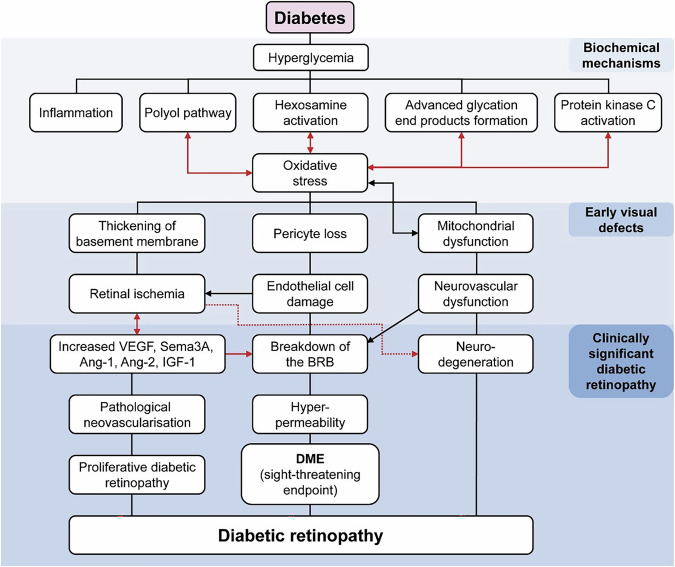
Pathogenesis and pathophysiology of DR*.* Adapted from Ansari et al. [26]. Additional links shown in red are based on publications by Kang et al. [27], Cerani et al. [28], Joyal et al. [17], Lechner et al. [2], Khanh Vu et al. [29] and Zhou et al. [30]. Adapted under the open-access Creative Commons Attribution license. Ang angiopoietin, DME diabetic macular edema, DR diabetic retinopathy, IGF-1 insulin-like growth factor 1, PDR proliferative DR, PKC protein kinase C, Sema3A semaphorin 3A, VEGF vascular endothelial growth factor.

Chronic hyperglycaemia triggers the initial clinical features of DR through changes to the vascular wall and microvascular damage; microvascular changes in DR include capillary blockages (which may involve leucostasis), increased vessel leakage and irregularities in blood flow (Fig. 2) [2, 17, 26–30]. Microvascular changes can result in inadequate blood flow to the metabolically active retina, leading to RNP and subsequent ischaemia [4]. Ischaemia-related damage to the retina and surrounding vasculature can initiate a cycle that perpetuates the progression of ischaemia by releasing various mediators (e.g. Sema3A) and can lead to complications [17, 31]. One such complication is DMI secondary to RNP, which can cause irreversible vision loss [4, 9]. Irreversible vision loss in DMI occurs due to extensive damage to both the retinal microvasculature and the neurosensory layer of the retina, which is an interconnected process [31]. The likelihood of DMI increases with the severity and duration of DR [31].

## The Sema3a/Nrp1 pathway in DR and RNP

In RNP secondary to DR, physiological angiogenesis is disrupted [17]. Initial damage to the retinal microvasculature and hypoxia are followed by the release of various factors by retinal glial cells (e.g. vascular endothelial growth factor [VEGF]) to drive physiological angiogenesis and aim to restore the metabolic equilibrium [17]. This mechanism was demonstrated in a murine model in which mouse pups were exposed to 75% oxygen from P7 to P12 to develop oxygen-induced retinopathy (OIR), followed by room air until P17 [23]. Relative to wild-type mice, mice with OIR had a peak in Sema3A messenger RNA levels at P12 (~7-fold increase) followed by a peak in VEGF messenger RNA levels at P14 (~6-fold increase) [23]. Increased Sema3A levels form a chemical barrier in the retina that directs new blood vessels away from the ischaemic retina, locally preventing physiological revascularisation [17] and contributing to retinal neurosensory and capillary damage that results in progressive vision loss [31]. This imbalance between VEGF and Sema3A is believed to dysregulate angiogenesis, guiding blood vessels away from ischaemic regions of the retina and into the vitreous cavity, leading to pathological retinal neovascularisation [17].

Sema3A is a guidance molecule that directs vessel growth by vasorepulsion [17]. It induces cytoskeletal collapse in the filopodia of endothelial tip cells, thus locally repelling them from the ischaemic retina [17]. Nrp1 has two extracellular ligand-binding domains, A and B, that respectively bind Sema3A and VEGF-A proteins [18]. Dysregulation of Sema3A and VEGF-A signalling can promote misguided pathological angiogenesis and hyperpermeability in the eye (Fig. 1) [17–20].

The role of the Sema3A/Nrp1 pathway in regulation of angiogenesis and vascular permeability has been indicated in preclinical models. In the murine OIR model, mice with Nrp1 hypomorphism (partial loss of function) have reduced pathological angiogenesis compared with wild-type mice [32]. An antibody against Nrp1 increased physiological revascularisation of avascular areas in the OIR model [33]. This shows redirection of angiogenesis towards the ischaemic retina and away from the vitreous cavity [33]. In addition, OIR mice lacking endothelial Nrp1 have reduced neovascularisation in the retina [34], and an antibody targeting Nrp1 reduced VEGF-induced retinal permeability in an Evans blue rat model [33]. In patients with PDR, Nrp1 was expressed in fibrovascular proliferative tissue surgically excised at vitrectomy, and Nrp1 co-expression with VEGF receptor 2 correlated with vascular density of the tissue, suggesting a potential role for Nrp1 in angiogenic activity in humans [35].

Elevated levels of Sema3A have been found in the plasma of patients with DR [36] and in the vitreous of patients with late-stage proliferative DR [23] and DMO [28]. Animal studies have shown that, under hypoxic conditions, retinal ganglion cells secrete Sema3A, and Sema3A blocks physiological revascularisation in the ischaemic retina [17]. In a murine model of OIR, silencing *Sema3A* gene expression enhanced physiological vascular regeneration in the ischaemic retina, thus inhibiting destructive vitreal neovascularisation and preserving neuroretinal function [17], suggesting that Sema3A may be a suitable therapeutic target for the treatment of DR.

## Impact of currently available therapies on RNP

In this section, the available evidence for the impact of current therapies for DR and DMO on RNP is discussed, including laser treatment, intravitreal (IVT) anti-VEGF agents and IVT corticosteroids.

Currently, there is no consensus on whether pan-retinal photocoagulation (PRP) impacts RNP apart from a known destructive effect on ischaemic cells, with older studies suggesting that PRP results in decreased retinal blood flow and more recent studies suggesting either no effect or increased perfusion when measured centrally [37]. In a prospective, observational, consecutive case series of 20 eyes from 15 patients with PDR treated with a single session of PRP and assessed with widefield OCTA, no change in RNP was observed immediately after PRP treatment, and RNP appeared to remain stable for up to 1 year [37]. However, differentiation of RNP from secondary effects of PRP, such as inflammation, is challenging. As such, further research is needed to confirm the treatment effects of PRP on RNP.

Ranibizumab and aflibercept are currently the only anti-VEGF agents approved for the treatment of DR with and without DMO [38–40]. Additionally, two other anti-VEGF agents, brolucizumab and faricimab, are approved for the treatment of DMO [38, 41, 42]. The impact of approved anti-VEGF agents on underlying RNP and ischaemia in patients with DR with or without DMO has been reviewed previously, with authors concluding that the results are conflicting and differ depending on the imaging modality used to assess RNP and the specific location of RNP [43, 44]. Generally, studies of anti-VEGF agents report improvements in Diabetic Retinopathy Severity Scale (DRSS) score and, if at all, only a limited impact on perfusion and disease progression [43, 44]. These studies include several *post hoc* and retrospective analyses of Phase III fluorescein angiography studies in participants with DMO, such as the RISE, RIDE, RESTORE and VISTA trials [45–47].

The major challenge is the lack of robust natural history studies about progression of RNP, and accordingly, reported potential treatment effects are difficult to differentiate from the natural history of RNP in DR. For example, some studies in PDR reported increases in RNP area after treatment with anti-VEGF agents; however, these changes are difficult to differentiate from the expected progression of RNP in this population [48–50]. In a prospective, randomised single-centre substudy of the larger CLARITY trial, 40 participants (40 eyes) with PDR were treated with either aflibercept or PRP and underwent mechanistic evaluation. Mean total area of RNP increased after 52 weeks in both the aflibercept (131.2–158.4 disc areas) and PRP groups (125.1–156.1 disc areas), with no statistically significant difference between groups [48]. Moreover, in the prospective, randomised RECOVERY trial, 40 participants with PDR and substantial RNP were randomised 1:1 to monthly or quarterly IVT aflibercept 2 mg [49, 50]. At 1 year of follow-up, the mean total RNP area on ultra-widefield fluorescein angiography imaging remained stable at 264 mm^2^ (p = 0.70) with monthly treatment but increased from 207 mm^2^ at baseline to 268 mm^2^ (p = 0.01) with quarterly treatment (p = 0.05, monthly vs quarterly) [50]. After 1 year of follow-up, patients switched to the alternative aflibercept schedule (i.e. from monthly to quarterly treatment or vice versa) [49]. The corresponding values at 2 years of follow-up were 386 mm^2^ with monthly treatment (p < 0.0001 vs baseline) and 421 mm^2^ with quarterly treatment (p < 0.0001 vs baseline; p = 0.023, monthly vs quarterly) [49].

In a recent meta-analysis of randomised controlled trials of 1296 eyes with 1 year of follow-up and 1131 eyes with 2 years of follow-up in patients with DR, RNP progression at both time points was slower among patients who received anti-VEGF therapy compared with macular laser therapy/PRP or sham [51]. Nonetheless, according to the Grading of Recommendations Assessment, Development and Evaluation guidelines, evidence was classified as ‘low’ due to indirectness and imprecision [51]. Taken together, these data suggest that anti-VEGF therapies may at best slow but not prevent RNP progression, with more frequent injections resulting in a stronger effect. However, it is important to highlight that the variable outcomes from these studies were likely influenced by the differences in the definition of RNP and the imaging modality used to assess it across studies [44, 51]. Differences in study design and patient populations may have also impacted these outcomes [48, 51]. For further evidence from larger randomised sham-controlled trials, development of standardised methodology for the assessment of RNP and a better understanding of the natural history of RNP are needed to fully understand the impact of anti-VEGF therapy on RNP at different stages of DR and to explore additional metrics to measure disease severity and impact.

The use of IVT corticosteroids in DMO, which is well established in the second line and in selected cases as first-line therapy [52], was reviewed by Rittiphairoj et al., who concluded that these agents effectively improved vision compared with sham or control in patients with DMO [53]. However, IVT corticosteroids are associated with an increased risk of cataract progression, an increased need for intraocular pressure‐lowering medications and, although rare, an increased need for glaucoma surgery, all of which can limit treatment benefits [53].

In general, the impact of IVT corticosteroids on DR has been less well investigated than that of anti-VEGF agents, although various IVT corticosteroids seem to be effective in preventing progression to vision-threatening complications of DR [54, 55]. Evidence on potential effects of corticosteroids on RNP is even more limited, as it is mainly based on few uncontrolled case series of the potential efficacy of IVT corticosteroids in patients with RNP secondary to DMO [56, 57]. As baseline DMO as well as the resolution of DMO in response to treatment is expected to have a substantial effect on measurement of RNP, data should be interpreted cautiously [58]. Although small-sample studies have evaluated perfusion status before and after administration of IVT corticosteroids, these studies lacked comparator arms and may have been subject to reporting bias [57, 59]. Some studies have reported reduced perfusion on OCTA with IVT corticosteroids [60, 61]. Therefore, while a slowdown of DR progression upon IVT corticosteroid treatment may be possible, it is difficult to draw robust conclusions on the effects of IVT corticosteroids on RNP owing to limited evidence from small studies and the lack of natural history data.

## Limitations of currently available therapies

Current standards of care for DR and its complications are invasive and include PRP and IVT anti‑VEGF agents [62].

PRP achieves regression of neovascularisation through the creation of thermal burns in the peripheral retina, ablating ischaemic retinal cells, enhancing retinal oxygenation and reducing VEGF release [38, 63]. PRP was established as the standard of care for PDR more than 40 years ago and has reduced the risk of severe vision loss by half [64, 65]. However, patients treated with PRP can experience loss of visual field [66] and dark adaptation [67], resulting in loss of ability to drive [68], and new-onset DMO [69]. In rare cases, PRP can burn other structures in the eye, including the lens and fovea [70, 71]. Worsened vision following PRP treatment, due to cystoid macular oedema or vitreous haemorrhage, can also occur [72]. Furthermore, many patients treated with PRP (>70%) experience moderate or high levels of pain [73], which are significantly higher than the pain levels experienced by patients treated with IVT injections [74]. As such, PRP can lead to the deterioration of patients’ perceived functional status, quality of life and treatment satisfaction [75].

Although IVT anti-VEGF agents are widely used to treat DMO [62], they are also becoming more popular for treating PDR without DMO in selected cases, and they have shown favourable outcomes on the DRSS and prevention of vision-threatening complications if administered at a sufficient frequency [76]. However, these agents have a high treatment burden, as intensive injection regimens may be needed due to a relatively short duration of action and limited impact on the underlying DR [39–41]. In addition, frequent, long-term treatment may be needed to maintain any initial improvements, and there is a risk of disease rebound if the injection interval is too long or injections are missed [77]. In the second year of the PANORAMA study, the proportion of patients who transitioned from aflibercept 2 mg every 8 weeks to as-needed dosing achieving a two-step or greater improvement in DRSS scores decreased from 79.9% of patients at Week 52 to 50.0% at Week 100, suggesting that a reduction in treatment frequency may increase the risk of disease progression [76]. In a *post hoc* analysis of the RIDE and RISE trials and their open-label extensions, more than 30% of eyes whose scores improved to mild-to-moderate NPDR (DRSS score ≤ 43) with ranibizumab treatment during RIDE/RISE experienced a one- to two-step worsening in DRSS scores during the open-label extension with less controlled and less frequent treatment regimens [78]. Patients with improved (mild-to-moderate) NPDR showed significant worsening in DRSS scores from open-label extension baseline to Month 48 compared with the native group between RIDE/RISE baseline and Month 12 (mean increase in DRSS score of 1.0 [95% CI 0.7–1.4] vs 0.1 [95% CI −0.1 to 0.4]; p < 0.0001) [78].

This is of particular concern, as missed visits are a relatively frequent finding among patients with DR [77]. Reasons for missed IVT injection appointments include the presence of comorbidities, personal and family reasons, or problems with a clinic, insurance or change of physician [79]. In a real-world study in patients with DR and DMO, more than one quarter of patients were lost to follow-up within the first year of treatment [80]. In another study, treatment breaks of >100 days have been reported in almost half of patients with DR and DMO, with the number of missed appointments correlating with a rising number of scheduled treatment visits [79].

Early treatment of severe or moderately severe NPDR with anti-VEGF agents may prevent progression to vision-threatening PDR [81]. Although early treatment reduces the risk of progression to DMO or PDR and improves the anatomical appearance of NPDR, it does not appear to benefit visual acuity [82]. As such, although it is sometimes difficult to justify the burden of regular IVT injections to patients, frequent long-term treatment may be required to attain optimal outcomes [82].

Due to the high treatment burden, compliance can also be low among patients with PDR receiving anti-VEGF treatment [66, 83]. In a small retrospective study, eyes with PDR lost to follow-up during IVT anti-VEGF monotherapy exhibited worse anatomic and functional outcomes than eyes receiving PRP [77]. In long-term trials, loss to follow-up is common (22–39% of patients) [66, 83] and has been associated with younger age, lower income, and race [83]. Adherence to follow-up for anti-VEGF IVT injections is critical for the effective management of DR and for maintaining visual outcomes in the long term; cessation of regular injections may result in disease worsening and, ultimately, irreversible vision loss [77]. Among patients lost to follow-up, mean visual acuity is significantly worse at the return and final visits compared with the visit before loss to follow-up, and there is a high incidence of tractional retinal detachment (10 of 30 patients) and neovascularisation of the iris (4 of 30 patients) [77].

Adverse events and complications of IVT anti-VEGF therapies have been extensively reviewed [38, 84]. Adverse events associated with anti-VEGF therapy are mainly injection-related and include cataracts, vitreous haemorrhage, uveitis and ocular inflammation, floaters, retinal vessel changes, retinal detachment, endophthalmitis and elevated intraocular pressure [38, 84]. Anti-VEGF therapy may also affect systemic VEGF levels, which may explain the suggested association of anti-VEGF treatments with systemic adverse events such as cardiovascular events [38, 84]; however, more research is needed to confirm these findings.

IVT anti-VEGF therapies have a negative effect on patients’ quality of life through the intensive injection regimen, effects on patients’ ability to work and absenteeism, and anxiety and discomfort [85]. Injection appointments, including travel time, have been estimated to take an average of 4.5 h, and the total injection appointment burden over 6 months has been estimated at 20 h per patient [85]. More than half of working patients (53%) need to use at least 1 day of holiday per appointment, and 71% of patients need a carer’s assistance at the time of the injection appointment [85]. Moreover, 75% of patients experience anxiety about their upcoming injection, with 54% reporting feelings of anxiety for at least 2 days before the injection [85]. These feelings of anxiety affect the ability to sleep well in 30% of patients, reduce concentration in 17% of patients, and can cause physical adverse events such as exhaustion [85].

## Novel agents targeting the Sema3a/Nrp1 pathway in ophthalmology

The Sema3A/Nrp1 pathway is considered a key driver of vascular guidance during physiological revascularisation in the ischaemic retina [16, 17, 86]. Thus, modulation of Sema3A and/or Nrp1 activity (i.e. by neutralising antibodies) may shift the balance between pro-angiogenic mediators, such as VEGF, and vasorepulsive mediators, such as Sema3A. This would redirect angiogenesis towards physiological revascularisation within the retina, thereby revascularising areas of RNP, and might modify one of the underlying pathophysiological causes of DR, leading to sustained improvement of DR and preservation of vision.

**Fig. 3:**
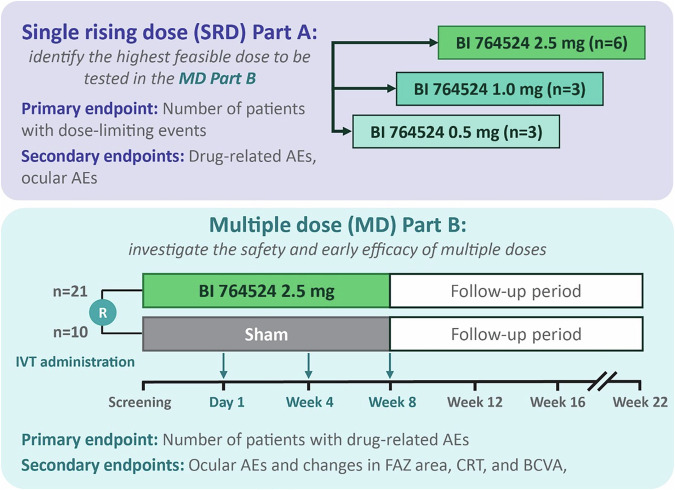
HORNBILL study design. Adapted from Chong et al. [87]. Adapted under the open-access Creative Commons Attribution license. AE adverse event, BCVA best corrected visual acuity, CRT central retinal thickness, D day, FAZ foveal avascular zone, IVT intravitreal, MD multiple dose, SRD single rising dose, W week.

One Phase I/IIa trial has investigated a Sema3A antibody (BI 764524; NCT04424290, HORNBILL; Fig. 3) in participants with PRP-treated DMI secondary to DR [87].

The trial comprised a non-randomised, open-label, single rising dose part and a randomised, masked, sham-controlled multiple-dose part to investigate the safety, tolerability and early biological responses to the IVT administration of Sema3A antibody in adults ≥18 years of age with DMI secondary to DR [87]. The primary endpoint of the single rising dose part was the number of patients with dose-limiting events until Day 8, and the primary endpoint of the multiple-dose part was the number of patients with drug-related adverse events from baseline to study end [87]. Secondary endpoints for assessment of early, preliminary efficacy included changes from baseline in size of the foveal avascular zone, best corrected visual acuity and central retinal thickness [87]. Results from this study will provide insight into the potential for targeting the Sema3A pathway for the treatment of ischaemia and, more broadly, RNP in DR.

There are several Phase II trials of other compounds underway or recruiting, which, based on preclinical data, have the potential to improve RNP in DR. SPECTRA (NCT05393284) is investigating the efficacy and safety of OPL-0401, an oral Rho kinase 1/2 inhibitor, in patients with NPDR or mild PDR [88, 89]. PER-001, a first-in-class, small-molecule endothelin receptor agonist [90], is being assessed in DR (NCT06003751) [91] and glaucoma (NCT05822245) [92]. Additionally, a novel frizzled class receptor 4 agonist, SZN-413, has shown promise in preclinical studies [93]. In murine models, UBX1325, a small-molecule B-cell lymphoma-extra large (Bcl-xL) inhibitor, was shown to reduce retinal vascular permeability, decrease the size of avascular areas and preserve retinal cell function [94, 95]. Phase II trials of IVT UBX1325 include BEHOLD (NCT04857996) in participants with DMO [96] and ENVISION (NCT05275205) in participants with neovascular age‑related macular degeneration [97]. At 48 weeks in both trials, treatment with UBX1325 resulted in vision maintenance or improvement and was well tolerated, with no cases of significant intraocular inflammation, retinal artery occlusion, endophthalmitis or vasculitis [96, 97]. MAGIC (NCT05681884) is evaluating the safety and efficacy in NPDR of faricimab, a humanised bispecific antibody binding to human angiopoietin 2 and VEGF, with a focus on assessing effects on RNP [98].

Another therapeutic approach under consideration for retinal ischaemia in age-related macular degeneration is the growth factors NVB001 and NVB002, which have been shown to promote the growth of new, non-leaking retinal blood vessels in murine models of ischaemic eye disease [99]. Further assessment of NVB001 is planned in toxicokinetic studies and early clinical trials in humans [99].

## Conclusions

Future efforts in DR should focus on developing treatments targeting the underlying causes rather than downstream complications of DR to achieve sustainable improvements, preserved retinal function and reduced treatment burden. Treatments addressing RNP hold some promise of achieving these goals. In DR, an imbalance between VEGF and Sema3A signalling may be an important driver of the progression of RNP and the dysregulation of angiogenesis, which guides blood vessels away from ischaemic retinal regions and into the vitreous cavity. Despite improving the DRSS score, current therapies for DR do not appear to substantially improve underlying RNP or ischaemia, are limited by invasiveness, are associated with a high treatment burden and/or have negative effects on patients’ quality of life. Improving retinal perfusion in DR through modulation of angiogenesis by targeting the Sema3A/Nrp1 pathway may improve areas of ischaemia and break the cycle of retinal damage. In addition, several other pathways, such as the *Wnt* signalling pathway [93, 100], Rho kinase signalling pathway [88], and Bcl-xL pathway [94, 95] have been targeted by emerging agents with potential to improve ischaemia and RNP. Reductions in RNP area may correspond with more sustainable improvements in DR severity and better preservation of function, and these agents may be associated with a reduced treatment burden compared with current treatment options. A review of the studies investigating the impact of current treatments on RNP, as well as of emerging therapies for RNP in DR, highlights the need for the standardisation of imaging biomarkers related to RNP. This may include consensus-building initiatives using expert input to define the parameters of biomarkers for RNP measurement [101], the validation of automated image analysis tools to reduce variability of measurements [102] and the development of established morphological endpoints for use in clinical trials. Furthermore, there is a need for larger natural history studies to assess the natural progression of RNP and its impact on DR progression and other patient-relevant endpoints. Beyond this, further interventional studies are needed to investigate the translation of promising preclinical and clinical data into tangible benefits for patients with DR.

## Supplementary information

Sema3A MoA Manuscript Supplementary Podcast
