# Correction: New targets in diabetic retinopathy: addressing limitations of current treatments through the Sema3A/Nrp1 pathway

**DOI:** 10.1038/s41433-025-03971-3

**Published:** 2025-11-07

**Authors:** Sobha Sivaprasad, Chui Ming Gemmy Cheung, Martin Gliem, Charles C. Wykoff, Nina Zippel, Susumu Ishida, Quan Dong Nguyen

**Affiliations:** 1 National Institute of Health Research Biomedical Research Centre, Moorfields Eye Hospital NHS Foundation Trust, London, UK; 2 University College London Institute of Ophthalmology, London, UK; 3 SingHealth Research, Singapore, Singapore; 4 Boehringer Ingelheim International GmbH, Ingelheim am Rhein, Germany; 5 Retina Consultants of Texas, Blanton Eye Institute, Houston Methodist Hospital, Houston, TX USA; 6 Boehringer Ingelheim Pharma GmbH & Co. KG, Biberach an der Riß, Germany; 7 Department of Ophthalmology, Faculty of Medicine and Graduate School of Medicine, Hokkaido University, Sapporo, Japan; 8 Byers Eye Institute, Stanford University School of Medicine, Palo Alto, CA USA

**Keywords:** Retinal diseases, Eye diseases

Correction to: *Eye* 10.1038/s41433-025-03835-w, published online 09 July 2025

In this article, the “Plain Language Summary” and “Method of Literature Search” have been omitted during the production process. They should read:

Method of literature search

Background literature was searched in PubMed using search terms such as ‘diabetic retinopathy’, ‘diabetic macular ischemia’, ‘diabetic macular edema’, ‘semaphorin 3a’, ‘neuropilin 1’, ‘retinal non-perfusion’, ‘vascular perfusion’, ‘anti-VEGF’, ‘corticosteroid’ and ‘laser photocoagulation’. Selected articles in English included the following publication types: Clinical Study; Clinical Trial; Clinical Trial, Phase I; Clinical Trial, Phase II; Clinical Trial, Phase III; Clinical Trial, Phase IV; Clinical Trial Protocol; Controlled Clinical Trial; Meta-Analysis, Randomised Controlled Trial; Review; and Systematic Review. Reference lists from the selected articles were also reviewed, from which relevant articles were manually included into the final list, in addition to extensive general background reading about the topic. Additionally, ClinicalTrials.gov and Google searches were performed to identify upcoming trials of treatments in DR with the potential to improve RNP.

Plain language summary

The retina is the light-sensing layer at the back of the eye. Damage to the retina can lead to eye diseases. Diabetes can cause reduced blood flow to the retina, damaging the retina and leading to vision loss. Eye disease is common in people with diabetes. This review discusses different drugs currently used to treat sight loss in people with diabetes. Taking some of these drugs can affect the quality of a person’s life. For example, the treatment may need to be given by regular injections into the eye and/or may have upsetting side effects. This review also talks about new drugs now being studied to improve blood flow to the retina and slow or stop sight loss in people with diabetes. Proteins called semaphorin 3A and neuropilin 1 regulate blood flow to the retina. New drugs have been made that work against these proteins. These drugs may help to increase blood flow to the retina. Several other drugs that may increase blood flow to the retina are also being studied in animals and humans.

The original article has been corrected.

